# *Clostridioides difficile* infections; new treatments and future perspectives

**DOI:** 10.1097/MOG.0000000000000989

**Published:** 2023-11-09

**Authors:** Charmaine Normington, Caroline H. Chilton, Anthony M. Buckley

**Affiliations:** a Healthcare Associated Infections Research Group, School of Medicine, Faculty of Health and Medicine, University of Leeds; b Leeds Teaching Hospital Trust, Leeds General Infirmary; c Microbiome and Nutritional Sciences Group, School of Food Science & Nutrition, Faculty of Environment, University of Leeds, Leeds, UK

**Keywords:** *Clostridioides difficile*, dysbiosis, microbiome therapeutics, phage therapy

## Abstract

**Purpose of review:**

As a significant cause of global morbidity and mortality, *Clostridioides difficile* infections (CDIs) are listed by the Centres for Disease Control and prevention as one of the top 5 urgent threats in the USA. CDI occurs from gut microbiome dysbiosis, typically through antibiotic-mediated disruption; however, antibiotics are the treatment of choice, which can result in recurrent infections. Here, we highlight new treatments available and provide a perspective on different classes of future treatments.

**Recent findings:**

Due to the reduced risk of disease recurrence, the microbiome-sparing antibiotic Fidaxomicin has been recommended as the first-line treatment for *C. difficile* infection. Based on the success of faecal microbiota transplantations (FMT) in treating CDI recurrence, defined microbiome biotherapeutics offer a safer and more tightly controlled alterative as an adjunct to antibiotic therapy. Given the association between antibiotic-mediated dysbiosis of the intestinal microbiota and the recurrence of CDI, future prospective therapies aim to reduce the dependence on antibiotics for the treatment of CDI.

**Summary:**

With current first-in-line antibiotic therapy options associated with high levels of recurrent CDI, the availability of new generation targeted therapeutics can really impact treatment success. There are still unknowns about the long-term implications of these new CDI therapeutics, but efforts to expand the CDI treatment toolbox can offer multiple solutions for clinicians to treat this multifaceted infectious disease to reduce patient suffering.

## INTRODUCTION

As the causative agent of *Clostridioides difficile* infection (CDI), the bacterium *C. difficile* is a Gram-positive, anaerobic spore forming pathogen of the gastrointestinal tract. As a toxin-mediated disease, CDI poses a significant burden to patients and healthcare systems globally [1,2]. CDI causes a wide range of symptoms, ranging from mild self-limiting diarrhoea to life-threatening complications such as pseudomembranous colitis and toxic mega-colon. Antibiotic treatment can fail to fully resolve the primary infection, resulting in the relapse of disease in up to 20% of cases [3]. *C. difficile* has been listed as an urgent threat by the Centre for Disease Control (CDC, USA) with an estimated 500 000 cases per year and 12 800 deaths with $1B attributable costs in the United States [4]. The economic impact of CDI also represents a significant burden in Europe, with costs to healthcare systems estimated at €3000M per year [5,6]. Risk factors for the development of primary CDI include age ≥65 years, antibiotic use, with increased risk for each extra antibiotic prescribed, and prior hospital admission [7]. 

**Box 1:**
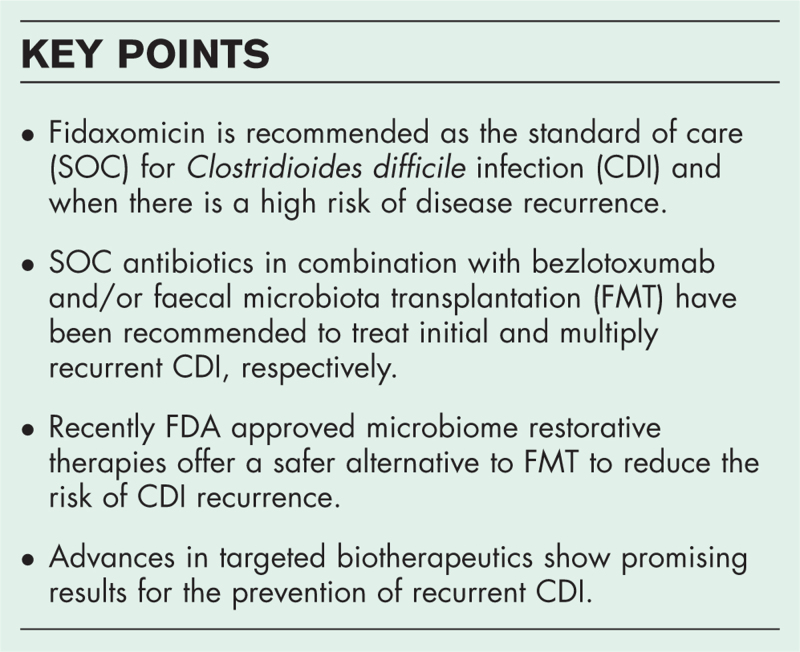
no caption available

## PATHOGENESIS

**FIGURE 1:**
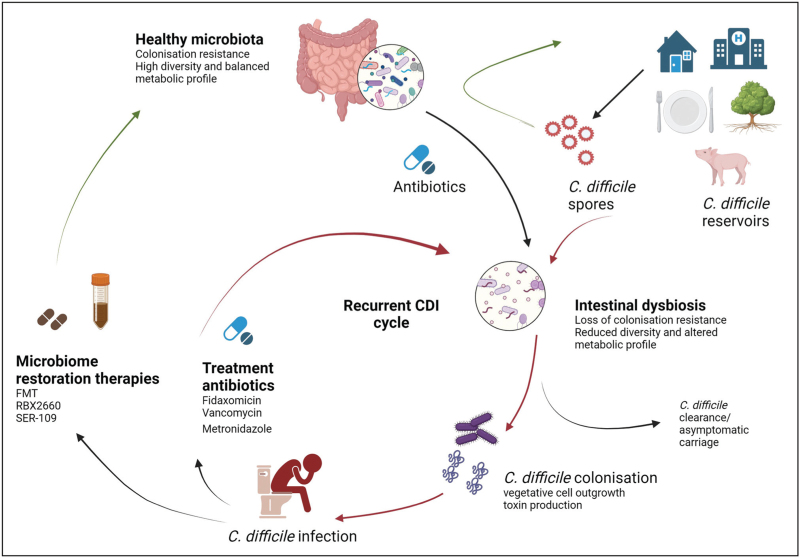
*C*. *difficile* infection and recurrence cycle. A healthy microbiota is refractive to *C. difficile* spore colonization from external reservoirs; however, disruption to the microbiota, especially through antibiotic exposure, can result in intestinal dysbiosis and the subsequent loss of colonization resistance. This creats an enviroment susceptible to *C. difficile* infection (CDI). Antibiotics used to treat CDI can perpetuate the intestinal dysbiosis and, in the presence on *C. difficile* spores, can lead to the recurrence of disease. A combination of antibiotics and microbiome restorative options have been shown to reduce the risk of disease recurrence. Created with BioRender.com.

CDI is mediated through the ingestion of spores. *C. difficile* spores are ubiquitous in the environment, especially healthcare settings, and are highly resistant to environmental pressures such as desiccation, extreme temperatures and standard disinfection procedures [8–10]. The ability of *C. difficile* spores to germinate and colonize the large intestine is largely dependent on the commensal microbiota and associated metabolome [11]. *C. difficile* spores remain quiescent until favourable conditions for germination; gut metabolite signals, such as primary bile acids and glycine, are potent germinators. These metabolite signals accumulate due to disruption in the microbiome-mediated bile acid metabolism pathways, typically through antibiotic consumption, which allows *C. difficile* spores to germinate and proliferate [12,13]. This facilitates the production of *C. difficile* toxins and leads to inflammation of the colonic membrane and subsequent symptoms of CDI (Fig. 1).

## CURRENT AND NEWLY APPROVED *Clostridioides difficile* INFECTION TREATMENTS

The vegetative bacteria can be successfully eliminated with antimicrobial therapy; however, the spores are highly resilient and can persist in the gut [14]. The treatment of initial CDI with antimicrobial therapy can cause further disruptions to the microbiota, exacerbating the intestinal dysbiosis and potentially leading to the relapse of symptoms, known as CDI recurrence. CDI recurrence is defined as the onset of symptoms within 8 weeks of clinical cure from a previous episode [15] and can occur in up to 20% of patients, with increasing frequency with each subsequent episode [16]. This highlights the need to combine current antimicrobial regimes with microbiota-restorative therapies to re-establish colonization resistance of the commensal microbiota and reduce the likelihood of recurrent disease.

In line with current guidelines, CDI treatment options include fidaxomicin, vancomycin and metronidazole [17,18]. Fidaxomicin is a narrow spectrum antibiotic that demonstrates superior preservation of the intestinal microbiota when compared to vancomycin and is associated with similar clinical cure rates and a lower risk of CDI recurrence [19–22]. Fidaxomicin standard therapy (200 mg, 12 hourly for 10 days) is recommended as the first choice for treatment of the initial episode of nonsevere CDI, severe CDI and severe-complicated/refractory CDI [17,18]. If fidaxomicin is not available, standard vancomycin therapy (125 mg, 6 hourly for 10 days) can be used as an alternative. Metronidazole is no longer recommended as a first line agent to treat CDI as the clinical cure rate demonstrated inferiority to vancomycin [23] and was found to be a robust predictor of recurrence [24]; however, despite recommendations, metronidazole remains a first line treatment option in many countries due to the cost and availability of alternatives [25–27].

## TREATMENT AND PREVENTION OF RECURRENCE

One notable change in the ESCMID treatment guidelines from previous editions is the treatment recommendations for patients at high risk of recurrence. Reducing recurrent CDI is a key goal that can be approached from a number of angles, including: reducing the population at risk of recurrence using effective infection control procedures and antimicrobial stewardship; re-establishing colonization resistance through intestinal microbiota restoration; increasing the patients’ immunity and disrupting the pathogenesis pathway.

## INTESTINAL MICROBIOTA RESTORATION

The microbiota plays a significant role in protecting the host from pathogen invasion through colonization resistance. The loss of colonization resistance, typically with antibiotic use, can create an environment susceptible to CDI. The use of antibiotics to treat CDI further exacerbates intestinal dysbiosis, leading to higher recurrence risk after each subsequent episode of recurrence [28]. The microbiota of patients with recurrent CDI is characterized by reduced community diversity [29] and altered metabolomes, with delayed and incomplete recoveries when compared to nonrecurring patients [30^▪▪^]. The restoration of the gut microbiome to a healthy state is essential for the prevention of recurrent CDI. This has led to the shift in treatment approaches with antibiotics used to target *C. difficile* directly followed by microbiota restoration therapy to replenish the microbiota and re-establish a metabolomic state consistent with a healthy gut (Fig. 1).

## FAECAL MICROBIOTA TRANSPLANTATION

Primary insights into the efficacy of gut microbiota restoration came from faecal microbiota transplantation (FMT) experiments, where the delivery of minimally manipulated faeces from a healthy donor to a recipient with recurrent CDI can restore colonization resistance leading to clinical cure rates of 76.1% [31]. The mechanisms underpinning the efficacy of FMT are largely unknown but likely to be multifactorial, combining the restoration of microbial diversity and the metabolic landscape [32]. Bile acids have been shown to affect *C. difficile* germination and vegetative cell growth; therefore, restoration of bile acid metabolism through the reconstitution of bile salt hydrolases has been implicated in FMT efficacy [33]. FMT has been recommended to treat multiply recurrent CDI (second or subsequent recurrence) after treatment with either fidaxomicin or vancomycin [17,25]. More recent studies have demonstrated a potential role for FMT in treating a first or second CDI episode [34^▪^], severe and severe-complicated CDI [35,36]. Despite the efficacy of FMT against recurrent CDI, safety concerns have been raised over the potential transmission of pathogens after the transfer of an extended-spectrum beta-lactamase (ESBL) producing *Escherichia coli* resulted in recipient fatalities [37]. With the gut microbiota being linked to other extra-intestinal diseases, the application of an undefined microbiota could have unknown long-term health implications, highlighting the need for standardized procedures for screening and processing and a move towards a more defined and characterized intervention. In 2022–2023, the US Food and Drug Administration authorized two first-in-class live biotherapeutics for the treatment of recurrent CDI.

## RBX2660 (REBYOTA)

FDA approved RBX2660, known commercially as REBYOTA, is a live biotherapeutic consisting of a consortium of microbes prepared from human faeces delivered as an enema. The faeces are subject to comprehensive and standardized pathogen screening and processing which is maintained as a frozen suspension. A randomized, double blinded, placebo-controlled phase III trial demonstrated superiority of RBX2660 in reducing recurrent CDI after antibiotic treatment when compared to a placebo (70.6% vs. 57.5% success rate, respectively) [38^▪▪^]. Metabolomic analysis revealed a shift from majority primary bile acids, as expected after antibiotic therapy, to predominantly secondary bile acids concurrent with RBX2660 therapy [39]. Treatment success rates, in both treatment and placebo arms, in this study could have been influenced by initial diagnostic assays, with PCR testing potentially leading to the inclusion of patients without active CDI. Additionally, patients were recruited after experiencing only one episode of recurrence and not multiply recurrent patients, which pose a far higher risk of additional recurrences.

## SER-109 (VOWST)

Another biotherapeutic to have been recently approved by the FDA is the microbiome therapeutic SER-109 (also known as Vowst). SER-109 is a consortium of viable purified Firmicutes spores that are administered orally over 3 consecutive days. In a double blind, placebo-controlled, randomized phase III trial, administration of SER-109 after standard antibiotic therapy was superior in reducing recurrence when compared to placebo (88% and 60% success rate, respectively) [40^▪▪^]. Success rates in a 24-week follow up were 78.7% and 52.7% for SER-109 and placebo, respectively [41]. Unlike RBX2660, SER-109 was administered to patients who had three or more episodes of CDI over the last 12 months with a positive toxin result at diagnosis, which would have increased the risk of recurrence and accounts for high recurrence rates in the placebo arm.

## INCREASING PATIENT IMMUNITY

Suboptimal immune responses to toxins produced by *C. difficile*, evidenced by low levels serum antibodies, are associated with increased risk of recurrence [42]. Bezlotoxumab (commercially known as Zinplava) is a human monoclonal antibody that binds to and subsequently neutralizes the effects *C. difficile* toxin B. In a phase 3 double-blind, randomized placebo-controlled trial, the addition of bezlotoxumab to standard antibiotic therapy significantly reduced CDI recurrence when compared to placebo (17% vs. 28%, respectively) [43]. Bezlotoxumab was approved by the FDA in 2016 and is now recommended as an adjunctive to vancomycin for initial treatment of patients with a high risk or recurrence or after a first recurrence [17,18]. More recently, bezlotoxumab has been shown to reduce CDI recurrence in immunocompromised patients [44] and shows promise in patients with ulcerative colitis [45]. Although effective at reducing the incidence of recurrence, there is a distinct lack of preventive options. *C. difficile* vaccines to prevent primary CDI would offer a huge economic benefit; however, vaccine candidates are currently limited [46,47] and the failure of a bivalent *C. difficile* toxoid vaccine [48] and the vaccine candidate VLA84 [47] on hold after phase 2 trials further compounds this [49].

## WHAT COULD FUTURE *Clostridioides difficile* INFECTION THERAPIES LOOK LIKE?

Here, we horizon scan potential therapies that could be used as CDI treatments, expanding the therapeutic toolbox in the fight to reduce CDI morbidity and mortality. This is not an exhaustive list, but highlights recent advances in this field.

## INTERRUPTING *C. difficile* PATHOGENESIS PATHWAYS

*C. difficile* spores are required for disease transmission and persistence, consequently, the sporulation pathway represents a significant therapeutic target. Mutations in Spo0A, a global regulator of sporulation initiation in *C. difficile*, resulted in a strain defective in spore formation that failed to persist in the environment and transmit disease [50]. Although a promising therapeutic target, further investigations revealed a subsequent increase in toxin production by various clinically relevant *C. difficile* strains [51], which highlights the need for caution when targeting early sporulation and focuses on the possibility that inhibition of late-stage sporulation could be a viable target mechanism. Another target in the pathogenesis pathway is the prevention of spore germination. A recent description of a novel oxadiazole antibiotic which exhibits bactericidal activity against *C. difficile* vegetative cells and inhibits spore germination [52] has demonstrated *in vivo* efficacy in a mouse model with reduced spore recovery and recurrence rates when compared to vancomycin.

## NEXT GENERATION LIVE BIOTHERAPEUTICS

Upcoming biotherapeutics providing an alternative to FMT therapy are composed of cultured bacteria instead of components derived from faecal material. Providing more defined and controlled production will mitigate many of the problems associated with FMT and faecal-derived products. VE303 (Vedanta Biosciences) is a defined consortium of eight nonpathogenic *Clostridium* spores cultured in cell banks and prepared under GMP regulations. Administered orally by capsule, the high dose regimen had a 13.8% recurrence incidence compared with a 45.5% recurrence rate seen in the placebo group [53^▪^]. Another method in the prevention of CDI is through direct competition with a nontoxigenic *C. difficile* strain (NTCD). *In vitro* models of the human gut have shown that prior inoculation with a NTCD strain successfully prevented the development of simulated CDI with the hypervirulent RT027 strain after administering a range of different antibiotics [54]. Recently, oral immunization of mice with spores from a genetically modified NTCD strain (NTCD_Tcd169) to target both *C. difficile* toxins and adhesion/colonization factors resulted in effective protection against hypervirulent strain RT027 R20291 and reduced excretion of R20291 spores when compared to treatment with NTCD strains [55]. NTCD-M3 (VP20621, Destiny Pharma), a drug candidate composed of spores of the nontoxin producing *C. difficile* strain M3, [56] significantly reduced recurrence rates in a Phase II trial with 11% recurrence rate in the VP20621 group compared with 30% in the placebo group [57].

## PHAGE THERAPY

The overuse of antibiotics and the emergence of multidrug resistant bacteria have created a global health problem and has caused researchers to shift their attention to alternative therapeutic options. One such option is the use of bacteriophages. Phage therapy provides a solution that would prevent intestinal dysbiosis associated with antibiotic use. However, the high specificity of phages pose their own problem with limited host range activity, relying on a phage cocktail to provide a broad coverage against the majority of clinically relevant *C. difficile* isolates. Recently, a promising candidate as an addition to a phage cocktail was identified. The phage ФCD1801 demonstrated a broad host range activity towards *C. difficile* ribotype 078 strain which has previously proved an elusive target [58^▪^]. Despite these advances, the majority of phages isolated with activity against *C. difficile* are temperate phages that integrate into the genome and have been associated with the transfer of antimicrobial resistance determinants through horizontal gene transfer [59]. They also have the additional problem of entering the lysogenic cycle and not killing the target cell, making the identification of lytic phages with *C. difficile* specificity a priority [60]. Recently, a potentially lytic phage of *C. difficile* was identified that could overcome the issue seen with lysogenic phages [61] and advances in synthetic biology have paved the way for creating lytic variants of temperate phages that could provide a possible answer [60].

## CONCLUSION

CDI is mediated through antibiotic-induced intestinal dysbiosis that is exacerbated by current recommended first-in-line antibiotic therapy options, leading to high rates of CDI recurrence. The success of FMT therapy for multiply recurrent CDI has paved the way for next generation live biotherapeutics. Therapies targeted at microbiome restoration as an adjunct to antibiotic therapy can significantly improve treatment success and reduce the risk of disease recurrence. However, evidence suggests survival of *C. difficile* spores within mucosal biofilm populations, which could have implications for extended treatment success [14], emphasizing the need for biofilm targeted therapies. The long-term implications of these new CDI therapeutics are still mostly unknown, but efforts to expand the options for CDI treatment can offer multiple alternative solutions to treat this multifaceted infectious disease.

## Acknowledgements

*None.*

### Financial support and sponsorship

*None.*

### Conflicts of interest

*A.B. has received financial support to attend meetings and research funding from Seres Therapeutics Inc., Motif Biosciences plc., Nabriva Therapeutics plc, Tetraphase Pharmaceuticals, Almirall SA, GlaxoSmithKline plc, and Hayashibara Co. Ltd. C.C. has received research funding from Da Volterra, Paratek Plc, Actavis, Teva Pharmaceuticals USA Inc., Astellas Pharma Europe Ltd, Cubist Pharmaceuticals, Seres Therapeutics, Debiopharm and has received an honorarium from Astellas Pharma Europe Ltd. C.N. has received research funding from Seres Therapeutics inc, Da Volterra and Debiopharm.*

## REFERENCES AND RECOMMENDED READING

Papers of particular interest, published within the annual period of review, have been highlighted as:

▪ of special interest
▪▪ of outstanding interest
